# Dysregulation of stress granule dynamics by DCTN1 deficiency exacerbates TDP-43 pathology in *Drosophila* models of ALS/FTD

**DOI:** 10.1186/s40478-024-01729-8

**Published:** 2024-02-04

**Authors:** Tetsuhiro Ueda, Toshihide Takeuchi, Nobuhiro Fujikake, Mari Suzuki, Eiko N. Minakawa, Morio Ueyama, Yuzo Fujino, Nobuyuki Kimura, Seiichi Nagano, Akio Yokoseki, Osamu Onodera, Hideki Mochizuki, Toshiki Mizuno, Keiji Wada, Yoshitaka Nagai

**Affiliations:** 1 Department of Neurology, Faculty of Medicine, Kindai University, 377-2 Ohnohigashi, Osakasayama, Osaka 589-8511 Japan; 2 Life Science Research Institute, Kindai University, 377-2 Ohnohigashi, Osakasayama, Osaka 589-8511 Japan; 3 Department of Neurotherapeutics, Osaka University Graduate School of Medicine, Osaka, 565-0871 Japan; 4 Department of Neurology, Osaka University Graduate School of Medicine, Osaka, 565-0871 Japan; 5 Department of Neurology, Kyoto Prefectural University of Medicine, Kyoto, 602-0841 Japan; 6 Department of Degenerative Neurological Diseases, National Institute of Neuroscience, National Center of Neurology and Psychiatry, Tokyo, 187-8502 Japan; 7 Department of Neurophysiology, National Institute of Neuroscience, National Center of Neurology and Psychiatry, Tokyo, 187-8502 Japan; 8 Department of Veterinary Associated Science, Faculty of Veterinary Medicine, Okayama University of Science, Ehime, 794-8555 Japan; 9 Department of Neurology, Brain Research Institute, Niigata University, Niigata, 951-8585 Japan

**Keywords:** TDP-43, DCTN1, Stress granule, Microtubule-dependent transport, Aggregation

## Abstract

The abnormal aggregation of TDP-43 into cytoplasmic inclusions in affected neurons is a major pathological hallmark of amyotrophic lateral sclerosis (ALS) and frontotemporal dementia (FTD). Although TDP-43 is aberrantly accumulated in the neurons of most patients with sporadic ALS/FTD and other TDP-43 proteinopathies, how TDP-43 forms cytoplasmic aggregates remains unknown. In this study, we show that a deficiency in DCTN1, a subunit of the microtubule-associated motor protein complex dynactin, perturbs the dynamics of stress granules and drives the formation of TDP-43 cytoplasmic aggregation in cultured cells, leading to the exacerbation of TDP-43 pathology and neurodegeneration in vivo. We demonstrated using a *Drosophila* model of ALS/FTD that genetic knockdown of DCTN1 accelerates the formation of ubiquitin-positive cytoplasmic inclusions of TDP-43. Knockdown of components of other microtubule-associated motor protein complexes, including dynein and kinesin, also increased the formation of TDP-43 inclusions, indicating that intracellular transport along microtubules plays a key role in TDP-43 pathology. Notably, DCTN1 knockdown delayed the disassembly of stress granules in stressed cells, leading to an increase in the formation of pathological cytoplasmic inclusions of TDP-43. Our results indicate that a deficiency in DCTN1, as well as disruption of intracellular transport along microtubules, is a modifier that drives the formation of TDP-43 pathology through the dysregulation of stress granule dynamics.

**Supplementary Information:**

The online version contains supplementary material available at 10.1186/s40478-024-01729-8.

## Introduction

The abnormal aggregation of proteins into inclusions in the central nervous system is a common pathological hallmark of neurodegenerative diseases, including Alzheimer’s disease, Parkinson’s disease, amyotrophic lateral sclerosis (ALS), and frontotemporal dementia (FTD). TAR DNA-binding protein 43 (TDP-43), a DNA/RNA-binding protein, is a major component of the cytoplasmic inclusions formed in the affected neurons of patients with ALS and FTD [1, 31]. Almost all ALS patients and half of all FTD patients are thought to have TDP-43 pathology; ALS and FTD are currently recognized as a spectrum disorder with common genetic, clinical, and pathological aspects [22]. TDP-43 is mainly localized in the nucleus, and TDP-43 in cytoplasmic inclusions is known to undergo various post-translational modifications, such as ubiquitination, phosphorylation, and truncation [41]. Cytoplasmic inclusions of TDP-43 are accompanied with the depletion of TDP-43 in the nucleus of affected neurons of ALS/FTD patients, and therefore, two major pathogenic mechanisms have been suggested; a gain of toxic function triggered by TDP-43 aggregation, and loss of the physiological functions of TDP-43 through its mislocalization [19, 20]. Because TDP-43 plays essential roles in cellular activities, including nucleocytoplasmic transport, RNA processing, and stress granule metabolism, the aggregation and mislocalization of TDP-43 are likely to cause abnormalities in various cellular functions, leading to detrimental consequences for cell survival [37].

Despite the central roles of TDP-43 in the pathogenesis of ALS/FTD, the mechanism of TDP-43 aggregation remains unclear. Upon stress, TDP-43 is known to physiologically form cytoplasmic condensates called stress granules [41]. Stress granules are cytoplasmic membraneless organelles that are transiently formed under stress conditions through the liquid–liquid phase separation of RNA-binding proteins, RNA, and other components, which are readily disassembled during recovery after stress. The carboxy-terminal domain of TDP-43 is intrinsically disordered with low complexity, and plays an important role in the formation of stress granules [4, 5]. Intriguingly, missense mutations in TDP-43 that are associated with familial cases of ALS/FTD are mostly located in the carboxy-terminal low-complexity domain, and are found to potentially increase the aggregation propensity of TDP-43. Therefore, these familial mutations may disrupt the physiological liquid–liquid phase separation and/or stress granule dynamics, triggering the pathological aggregation of TDP-43 in the cytoplasm [21]. However, in most sporadic ALS/FTD patients, wild-type TDP-43 without mutations is aggregated into inclusions, and thus, it is unclear as to what drives the aggregation of wild-type TDP-43 in sporadic ALS/FTD.

The abnormal aggregation of TDP-43 is also observed in other neurodegenerative diseases, including Perry syndrome [27, 44] and inclusion body myopathy associated with Paget disease of bone and frontotemporal dementia (IBMPFD) [30, 33], which are referred to as secondary TDP-43 proteinopathies. Perry syndrome is an autosomal dominant neurodegenerative disease, and TDP-43-positive neuronal inclusions have been observed in the substantia nigra, globus pallidus, and brainstem of patient brains [39]. Several missense mutations in DCTN1, a subunit of the microtubule-associated motor protein complex dynactin, have been identified as the genetic cause of Perry syndrome [9]. Because these mutations are mostly located in the microtubule-binding domain at the amino terminus, the functional loss of DCTN1 may play a key role in the formation of cytoplasmic TDP-43 inclusions [43]. Intriguingly, DCTN1 expression is markedly downregulated in motor neurons of sporadic ALS patients [18], and the missense mutation in DCTN1 has also been linked to the autosomal dominant distal hereditary motor neuronopathy type VIIB (HMN7B) [32], indicating the crucial role of DCTN1 not only in Perry syndrome, but also in motor neuron diseases, including ALS. Because intracellular transport along microtubules has been implicated in the formation and disassembly of stress granules [24], we hypothesized that DCTN1 dysfunction and/or impaired microtubule transport may cause abnormalities in stress granule dynamics, which potentially leads to the pathological aggregation of TDP-43 in the cytoplasm.

To test this hypothesis, in this study we investigated whether deficiency in DCTN1 and intracellular transport along microtubules drives the formation of cytoplasmic TDP-43 aggregates in vivo. We demonstrated using a *Drosophila* model of ALS/FTD that genetic knockdown of DCTN1, as well as other components of microtubule-associated motor protein complexes, accelerates the formation of ubiquitin-positive cytoplasmic inclusions of TDP-43, leading to the exacerbation of neurodegeneration. Notably, DCTN1 knockdown delayed the disassembly of stress granules in stressed cells, leading to an increase in the formation of pathological cytoplasmic inclusions of TDP-43. These results demonstrate that DCTN1 and other microtubule-associated motor proteins are modifiers that drive the aggregation of wild-type TDP-43 through the dysregulation of stress granule dynamics, indicating the crucial role of intracellular transport along microtubules in the pathological development of TDP-43 proteinopathies, including ALS/FTD.

## Results

### DCTN1 knockdown exacerbates TDP-43-mediated neurodegeneration in ALS/FTD flies

**Fig. 1:**
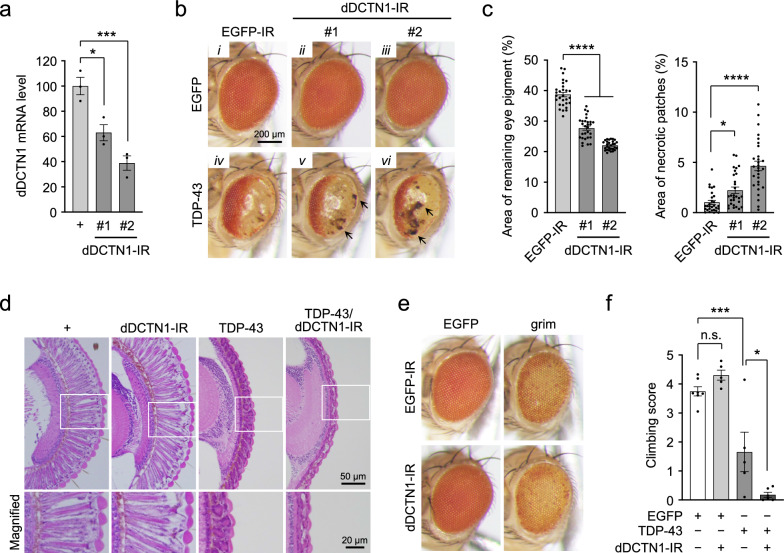
DCTN1 knockdown exacerbates TDP-43 toxicity in *Drosophila.* **a** Relative levels of dDCTN1 mRNA in adult fly brains analyzed by quantitative RT-PCR. Data were normalized using *rp49* mRNA levels, and are presented as relative values to dDCTN1 mRNA levels of wild-type flies. Data are presented as the mean ± standard error of the mean (SEM) of three independent experiments. Each dot represents data from a single experiment. Fly genotypes: +, *Elav-Gal4*/+; dDCTN1-IR#1, *Elav-Gal4*/+;*dDCTN1-IR#1*/+; dDCTN1-IR#2, *Elav-Gal4*/*dDCTN1-IR#2*. **b** Light microscopic images of compound eyes of EGFP flies or TDP-43 flies coexpressing EGFP-IR or dDCTN1-IR (#1 or #2) under the control of the GMR-Gal4 driver. Arrows indicate necrotic patches. Scale bar, 200 μm. **c** Bar graphs showing the area of remaining eye pigment (left) and the area of necrotic patches (right) of TDP-43 flies in **b**. Each dot represents data from each fly (*n* = 24–26 flies). Fly genotypes: EGFP/EGFP-IR, *GMR-Gal4*/+;*EGFP*/+;*EGFP-IR*/+; EGFP/dDCTN1-IR#1, *GMR-Gal4*/+;*EGFP*/*dDCTN1-IR#1*; EGFP/dDCTN1-IR#2, *GMR-Gal4*/*dDCTN1-IR#2*;*EGFP*/+; TDP-43/EGFP-IR, *GMR-Gal4*/+;;*TDP-43*/*EGFP-IR*; TDP-43/dDCTN1-IR#1, *GMR-Gal4*/+;*dDCTN1-IR#1*/+;*TDP-43*/+; TDP-43/dDCTN1-IR#2, *GMR-Gal4*/*dDCTN1-IR#2*;;*TDP-43*/+. **d** Histological analysis of eye sections of adult flies. Scale bars, 50 µm (top) and 20 µm (bottom). Fly genotypes: +, *GMR-Gal4*/+; dDCTN1-IR, *GMR-Gal4*/+;*dDCTN1-IR#1*/+; TDP-43, *GMR-Gal4*/+;;*TDP-43*/+; TDP-43/dDCTN1-IR, *GMR-Gal4*/+;*dDCTN1-IR#1*/+;*TDP-43*/+. The bottom images are magnified images of the boxed regions. **e** Light microscopic images of compound eyes of EGFP flies or grim flies coexpressing EGFP-IR or dDCTN1-IR#1 under the control of the GMR-Gal4 driver. Fly genotypes: EGFP/EGFP-IR, *GMR-Gal4*/+;*EGFP*/+;*EGFP-IR*/+; EGFP/dDCTN1-IR, *GMR-Gal4*/+;*EGFP*/*dDCTN1-IR#1*; grim/EGFP-IR, *GMR-Gal4*/+;*GMR-grim*/+;*EGFP-IR*/+; grim/dDCTN1-IR#1, *GMR-Gal4*/+;*GMR-grim*/*dDCTN1-IR#1*. **f** Bar graph showing climbing scores of EGFP or TDP-43 flies coexpressing dDCTN1-IR in the nervous system at 7 days of age. Data are presented as the mean ± SEM of 4 to 6 independent experiments. Each dot represents data from a single experiment. Fly genotypes: EGFP, *Elav-Gal4*/+;*EGFP*/+; EGFP/dDCTN1-IR, *Elav-Gal4*/+;*EGFP*/*dDCTN1-IR#1*; TDP-43, *Elav-Gal4*/+;;*TDP-43*/+; TDP-43/dDCTN1-IR, *Elav-Gal4*/+;*dDCTN1-IR#1*/+;*TDP-43*/+. Statistical analyses in **a**, **c**, and **f** were performed to assess differences from control flies by one-way ANOVA followed by the Dunnett multiple comparison test (*p < 0.05, ***p < 0.001, ****p < 0.0001) (**a**, **c**), or to assess differences among groups by one-way ANOVA followed by the Tukey multiple comparison test (*p < 0.05, ***p < 0.001; n.s., not significant) (**f**)

To investigate the pathological roles of DCTN1 deficiency in TDP-43 proteinopathy in vivo, we analyzed whether DCTN1 knockdown affects TDP-43-mediated neurodegeneration in a *Drosophila* model of ALS/FTD expressing human wild-type TDP-43. For DCTN1 knockdown, we used two transgenic fly lines expressing inverted repeat (IR) RNA against the *Drosophila* DCTN1 (dDCTN1) gene (dDCTN1-IR #1 and #2). Quantitative polymerase chain reaction (PCR) analysis showed that the levels of dDCTN1 mRNA are decreased by 37% (dDCTN1-IR #1) and 61% (dDCTN1-IR #2) compared with the control flies, confirming substantial knockdown of DCTN1 by both IR lines (Fig. 1a). Flies expressing TDP-43 under the control of the eye-specific GMR-Gal4 driver showed progressive degeneration of photoreceptor neurons and glial pigment cells, leading to the loss of pigmentation in the compound eyes (Fig. 1b, *iv vs. i*). Surprisingly, the coexpression of dDCTN1-IR (both #1 and #2 lines) in the TDP-43 flies resulted in a further decrease in eye pigmentation, together with the increased appearance of necrotic patches, compared with the TDP-43 flies coexpressing control EGFP-IR (percent area of remaining eye pigment: 27.7% ± 0.7% and 22.1% ± 0.3% for dDCTN1-IR #1 and #2, respectively, *vs.* 38.7% ± 0.8% for control EGFP-IR; percent area of necrotic patches: 2.23% ± 0.31% and 4.65% ± 0.48% for dDCTN1-IR #1 and #2, respectively, *vs.* 1.02% ± 0.20% for control EGFP-IR) (Fig. 1b, *v–vi vs. iv*, c). Similarly, heterozygous deletion of the dDCTN1 gene by *Df(3L)fz-GF3b* [13], a chromosomal deletion line that is deficient in various genes, including the dDCTN1 gene, also exacerbated eye degeneration in the TDP-43 flies (Additional file 1: Fig. SI-1). In contrast, dDCTN1-IR expression itself, as well as a heterozygous null mutation of the dDCTN1 gene, had no detrimental effects on eye size, pigmentation, and morphology in the control EGFP flies (Fig. 1b, *ii-iii vs. i*, and Additional file 1: Fig. SI-1b). Histological analysis revealed degeneration of photoreceptor neurons and pigment cells in the compound eyes of flies expressing TDP-43, and this TDP-43-mediated degeneration was exacerbated by the coexpression of dDCTN1-IR; almost complete loss of retinal structures was observed in the flies coexpressing TDP-43 and dDCTN1-IR, whereas the flies expressing dDCTN1-IR alone showed abnormalities only in the internal structure of the retina, i.e., small vacuole-like structures in the cornea, probably resulting from the toxicity induced by dDCTN1 reduction (Fig. 1d). Intriguingly, the coexpression of dDCTN1-IR had no effects on the apoptotic cell death induced by grim (Fig. 1e), suggesting that the effect of dDCTN1 knockdown on eye degeneration is specific to TDP-43 flies. These results indicate that dDCTN1 knockdown exacerbates the eye degeneration caused by TDP-43. We also investigated whether locomotor dysfunction caused by pan-neuronal expression of TDP-43 using the Elav-Gal4 driver is affected by dDCTN1 knockdown. The climbing assay demonstrated that flies expressing TDP-43 in the nervous system show a substantial decline in locomotor function at 7 days of age, which is further exacerbated by the coexpression of dDCTN1-IR (Fig. 1f). These results collectively indicate that DCTN1 deficiency exacerbates TDP-43-mediated toxicity in vivo.

### DCTN1 knockdown leads to the formation of ubiquitin-positive inclusions of TDP-43 in ALS/FTD flies

**Fig. 2:**
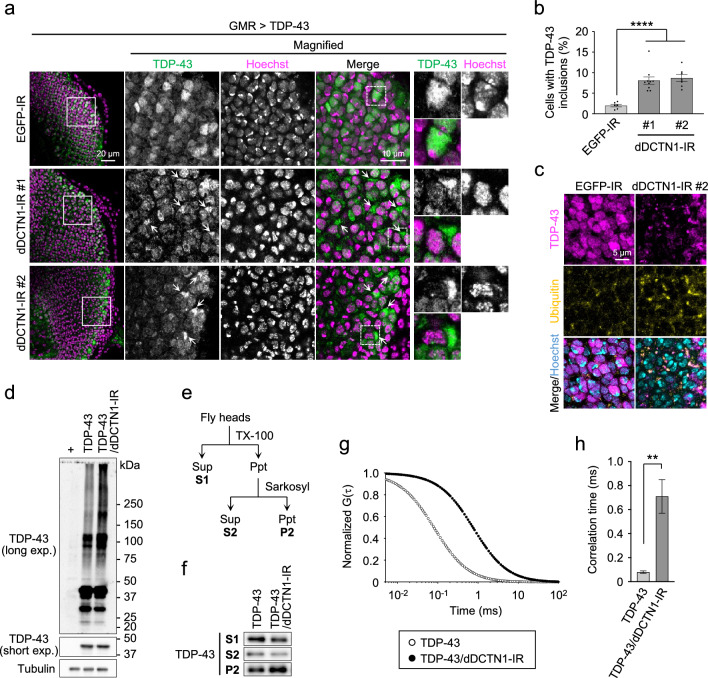
DCTN1 knockdown leads to the formation of ubiquitin-positive inclusions of TDP-43 in *Drosophila.* **a** Confocal microscopic images of TDP-43 antibody staining of larval eye discs of TDP-43 flies coexpressing EGFP-IR or dDCTN1-IR (#1 or #2). Arrows indicate cytoplasmic inclusions of TDP-43. Hoechst 33342 was used for nuclear staining. Scale bars, 20 µm (left) and 10 μm (right). The panels on the right are magnified images of the boxed region in the left panel. **b** Bar graph of the ratio of cells with cytoplasmic inclusions of TDP-43 shown in **a**. Data are presented as the mean ± SEM of 7–10 fly larvae. Statistical analysis was performed to assess differences from the EGFP-IR-expressing TDP-43 flies by one-way ANOVA followed by the Dunnett multiple comparison test (****p < 0.0001). **c** Confocal microscopic images of TDP-43 and ubiquitin antibody staining of larval eye discs of TDP-43 flies coexpressing EGFP-IR or dDCTN1-IR#2. Hoechst 33342 was used for nuclear staining. Scale bar, 5 μm. Fly genotypes: TDP-43/EGFP-IR, *GMR-Gal4*/+;;*TDP-43*/*EGFP-IR*; TDP-43/dDCTN1-IR#1, *GMR-Gal4*/+;*dDCTN1-IR#1*/+;*TDP-43*/+; TDP-43/dDCTN1-IR#2, *GMR-Gal4*/*dDCTN1-IR#2*;;*TDP-43*/+. **d** Western blot analysis of head lysates using antibodies against TDP-43. Smear bands with a molecular weight of more than 75 kDa were detected, and increased in the flies coexpressing dDCTN1-IR. **e** Schematic representation of the fractionation of fly head lysates using Triton X-100 and sarkosyl buffers. **f** Immunoblot images of fly head lysates fractionated by detergents. Fly genotypes: +, *GMR-Gal4*/+; TDP-43, *GMR-Gal4*/+;;*TDP-43*/+; TDP-43/dDCTN1-IR, *GMR-Gal4*/+;*dDCTN1-IR#1*/+;*TDP-43*/+. **g**, **h** Autocorrelation curves (**g**) and bar graph showing correlation time (**h**) obtained from FCS analysis of head lysates of TDP-43 flies. Fly genotypes: TDP-43, *GMR-Gal4*/+ ;*TDP-43-EGFP*/+ ; TDP-43/dDCTN1-IR, *GMR-Gal4*/+;*dDCTN1-IR#1*/+;*TDP-43-EGFP*/+

To investigate how DCTN1 deficiency exacerbates TDP-43-mediated toxicity in vivo, we performed immunohistochemical analysis of TDP-43 in TDP-43 flies coexpressing dDCTN1-IR. Confocal microscopic observation of larval eye discs from TDP-43 flies coexpressing control EGFP-IR showed that TDP-43 is mostly localized in the nucleus of photoreceptor neurons (Fig. 2a). The coexpression of dDCTN1-IR, however, led to a robust change in the cellular distribution of TDP-43; TDP-43 was less localized in the nucleus but mislocalized to the cytoplasm, and formed abnormal cytoplasmic inclusions in TDP-43 flies coexpressing dDCTN1-IR (Fig. 2a). Quantitative analysis confirmed that the number of cells with cytoplasmic TDP-43 inclusions is substantially increased in both dDCTN1-IR lines (#1 and #2) (Fig. 2b). Importantly, most of the TDP-43 inclusions formed in the cytoplasm were costained with the ubiquitin antibody (Fig. 2c). These results indicate that DCTN1 deficiency facilitates the formation of ubiquitin-positive cytoplasmic inclusion of TDP-43 in vivo, which recapitulates the characteristics of TDP-43 pathology in patients with ALS/FTD.

We next performed biochemical analyses to determine whether DCTN1 deficiency affects the expression level of TDP-43. Total proteins were extracted from head homogenates of the TDP-43 flies, and were subjected to Western blot analysis. However, there was no apparent difference in the protein levels of TDP-43 in the flies coexpressing TDP-43 and dDCTN1-IR compared with those expressing TDP-43 alone (Fig. 2d, short exp.), indicating no substantial effects of DCTN1 knockdown on TDP-43 expression in our flies. We then investigated whether DCTN1 deficiency affects the solubility of TDP-43. For this purpose, proteins extracted from the head lysates were fractionated by the difference in their solubility to detergents, including Triton X-100 and sarkosyl (Fig. 2e). Western blot analysis of the fractionated lysates demonstrated that the coexpression of dDCTN1-IR in TDP-43 flies led to an increase in sarkosyl-insoluble TDP-43 (Fig. 2f, fraction P2), but to a decrease in detergent-soluble TDP-43 (Fig. 2f, fractions S1 and S2), indicating that DCTN1 deficiency increases the insolubility of TDP-43 in vivo. We also found that the coexpression of dDCTN1-IR results in an increase in smear staining of TDP-43 with a molecular weight of more than about 75 kDa (Fig. 2d, long exp.), implying that the formation of high-molecular-weight species of TDP-43, or oligomeric assemblies of TDP-43, might be promoted by DCTN1 deficiency. In support of this, fluorescence correlation spectroscopy (FCS) analysis of flies expressing EGFP-tagged TDP-43 (TDP-43-EGFP) showed a rightward shift in the autocorrelation curve, indicating an increase in the correlation time in flies coexpressing dDCTN1-IR (correlation time: 0.71 ± 0.14 ms for TDP-43-EGFP/dDCTN1-IR *vs.* 0.08 ± 0.01 ms for TDP-43-EGFP) (Fig. 2g, h). Because correlation time is positively correlated with the apparent molecular size, the increase in correlation time in the TDP-43-EGFP flies coexpressing dDCTN1-IR suggests the formation of large assemblies of TDP-43. These results collectively indicate that DCTN1 deficiency leads to the formation of insoluble cytoplasmic aggregates of TDP-43 in vivo.

### DCTN1 knockdown delays the disassembly of stress granules in cells

**Fig. 3:**
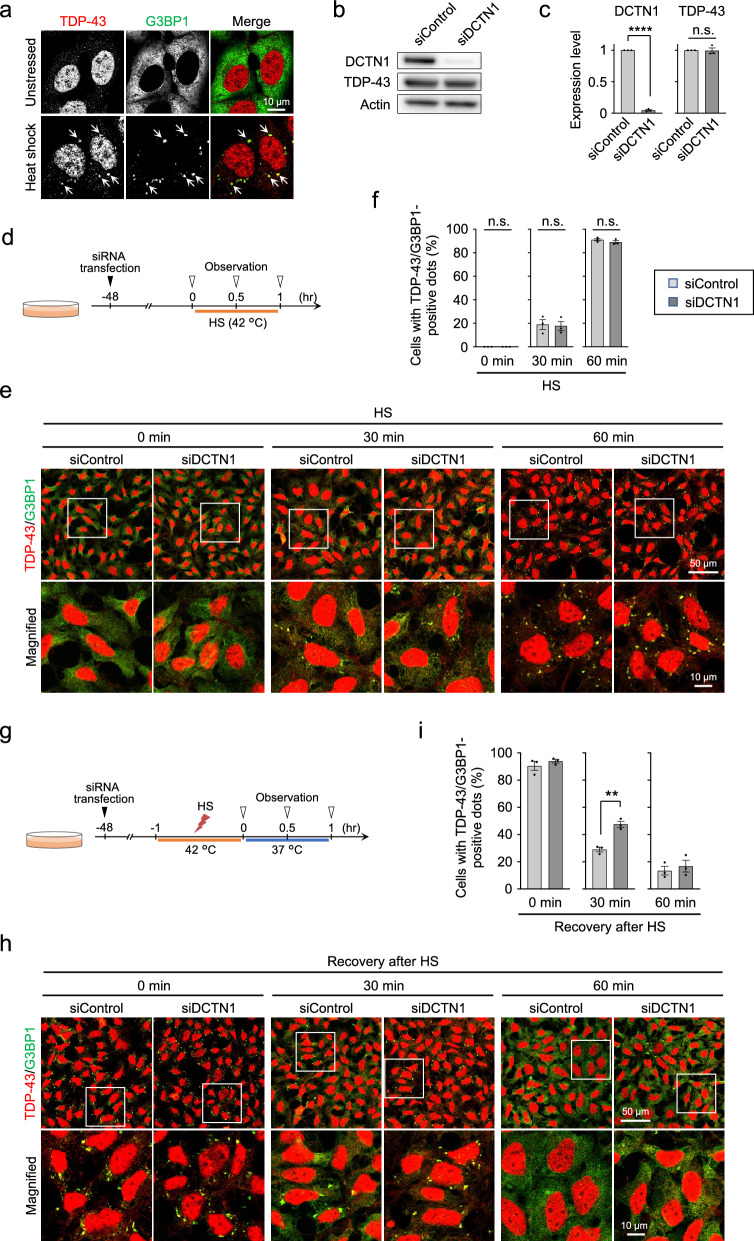
DCTN1 knockdown delays the disassembly of stress granules in heat-stressed cells. **a** Microscopic images of HEK293 cells after heat shock (HS) at 42 °C for 60 min. Upon heat stress, TDP-43 is colocalized with G3BP1, a marker of stress granules. Arrows indicate stress granules. **b**, **c** Western blot images (**b**) and bar graphs (**c**) showing relative expression levels of DCTN1 and TDP-43 in HEK293 cells that were transfected with an siRNA against DCTN1 (siDCTN1) or a non-targeting control siRNA (siControl). **d** Schematic representation of the HS experiments. **e**, **f** Microscopic images (**e**) and bar graphs (**f**) of stress granule formation after 30-min and 60-min HS. In (**e**), the panels at the bottom are magnified images of the boxed region in the top panels. In (**f**), the ratio of cells with cytoplasmic granules that are positive for both TDP-43 and G3BP1 were calculated. **g** Schematic representation of the recovery experiments after HS. **h**, **i** Microscopic images (**h**) and bar graphs (**i**) of stress granules analyzed at the recovery time of 30-min and 60-min after HS. Statistical analyses in **c**, **f**, and **i** were performed to assess differences between groups by the Student *t*-test (**p < 0.01, ****p < 0.0001; n.s., not significant). Scale bars, 10 µm (**a**), and 50 µm (top) and 10 µm (bottom) (**e**, **h**)

To elucidate as to how DCTN1 deficiency exacerbates cytoplasmic TDP-43 aggregation, we next performed cell culture experiments to investigate whether DCTN1 knockdown affects stress granule dynamics, which is likely regulated via microtubule-dependent transport. We confirmed that heat stress at 42 °C for 60 min leads to the formation of cytoplasmic granules that are stained by G3BP1, a marker protein of stress granules, in HEK293 cells (Fig. 3a). Cytoplasmic TDP-43 was mostly colocalized in these granules under the condition of heat stress (Fig. 3a). We also confirmed that the transfection of an siRNA against DCTN1 (siDCTN1) leads to a substantial decrease in DCTN1 protein level but not in TDP-43 protein level (Fig. 3b, c). Confocal microscopic analysis demonstrated that TDP-43-positive stress granules are formed in HEK293 cells that are transfected with control siRNA (siControl) after heat stress (18.9% ± 4.2% and 91.1% ± 1.1% for after 30-min and 60-min heat stress, respectively) (Fig. 3d–f). RNAi-mediated knockdown of DCTN1 using siDCTN1, however, showed no substantial changes in the efficiency of granule formation (ratio of stress granule-positive cells: 17.7% ± 4.0% and 89.0% ± 1.4% for 30-min and 60-min heat stress, respectively) (Fig. 3e, f), indicating that DCTN1 has only a minor role, if any, in the formation process of TDP-43-positive stress granules.

We next investigated the effects of DCTN1 knockdown on the disassembly of stress granules (Fig. 3g). For this purpose, HEK293 cells were subjected to heat stress at 42 °C for 60 min, similarly to the above experiment, and were then recovered at 37 °C after heat shock. Microscopic observation demonstrated that, although TDP-43-positive stress granules are formed in most of the siControl-transfected cells just after heat stress (at 0-min recovery), the ratio of cells with these granules are robustly decreased with time (ratio of stress granule-positive cells: 90.5% ± 3.3%, 29.1% ± 1.6%, and 13.5% ± 5.5% for 0-min, 30-min, and 60-min recovery after heat stress, respectively) (Fig. 3h, i), indicating that the disassembly of TDP-43-positive stress granules is a tightly regulated process that occurs rapidly after the removal of stress. Surprisingly, RNAi-mediated knockdown of DCTN1 resulted in an increase in the ratio of cells with TDP-43-positive stress granules at 30-min recovery after heat stress (ratio of stress granule-positive cells: 47.5% ± 2.2% for siDCTN1-transfected cells *vs.* 29.1% ± 1.6% for siControl-transfected cells) (Fig. 3h, i). These data indicate that DCTN1 knockdown delays the disassembly of TDP-43-positive stress granules, although this delay was not evident at 60-min recovery after heat stress (Fig. 3h, i). Intriguingly, a delay in the disassembly process of TDP-43-positive stress granules was also observed in cells treated with nocodazole, a microtubule depolymerization agent (Additional file 1: Fig. SI–2), indicating that a microtubule-dependent process acts as a key regulator in the disassembly of TDP-43-positive stress granules.

### DCTN1 knockdown increases TDP-43 aggregation in heat-stressed cells

**Fig. 4:**
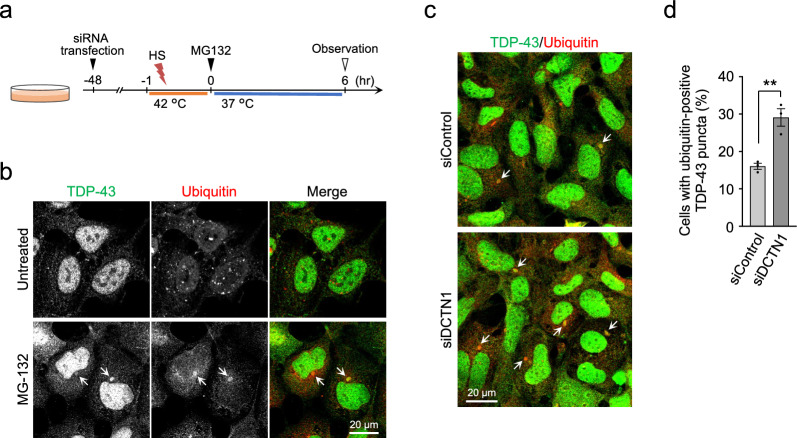
DCTN1 knockdown promotes the formation of ubiquitin-positive TDP-43 inclusions in heat-stressed cells. **a** Schematic representation of the experiment. HEK293 cells that were transfected with non-targeting control siRNA or DCTN1 siRNA were heat-shocked at 42 °C for 1 h, and then incubated at 37 °C for 6 h in the presence or absence of MG-132. **b** Microscopic images of HEK293 cells that were treated with MG-132 for 6 h after HS. **c**, **d** Microscopic images (**c**) and a bar graph (**d**) showing cytoplasmic TDP-43 inclusions costained with the ubiquitin antibody. Arrows in **b** and **c** indicate ubiquitin-positive inclusions of TDP-43 in the cytoplasm. Statistical analysis was performed to assess differences between groups by the Student *t*-test (**p < 0.01). Scale bar, 20 µm (**b**, **c**)

We next investigated whether DCTN1 knockdown affects the formation of TDP-43 aggregates. Previous studies have reported that the ubiquitin–proteasome system plays an important role in the clearance of stress granule components, and that impairment of proteasomal degradation leads to the aberrant accumulation of TDP-43 aggregates in the cytoplasm [34, 40, 42]. We confirmed that treatment of cells with the proteasome inhibitor MG-132 for 6 h after heat stress results in the formation of cytoplasmic inclusions of TDP-43 that are costained by a ubiquitin antibody (Fig. 4a, b), which is reminiscent of human TDP-43 pathology. Importantly, the ratio of cells with ubiquitin-positive TDP-43 inclusions was substantially increased by RNAi-mediated knockdown of DCTN1 (29.1% ± 2.3% for siDCTN1-transfected cells *vs.* 16.0% ± 0.8% for siControl-transfected cells) (Fig. 4c, d). These results indicate that DCTN1 knockdown leads to an increase in the formation of cytoplasmic TDP-43 aggregates under heat stressed conditions, possibly through dysregulation of the disassembly process of stress granules.

### Knockdown of microtubule-associated motor proteins exacerbates TDP-43 pathology in ALS/FTD flies

**Fig. 5:**
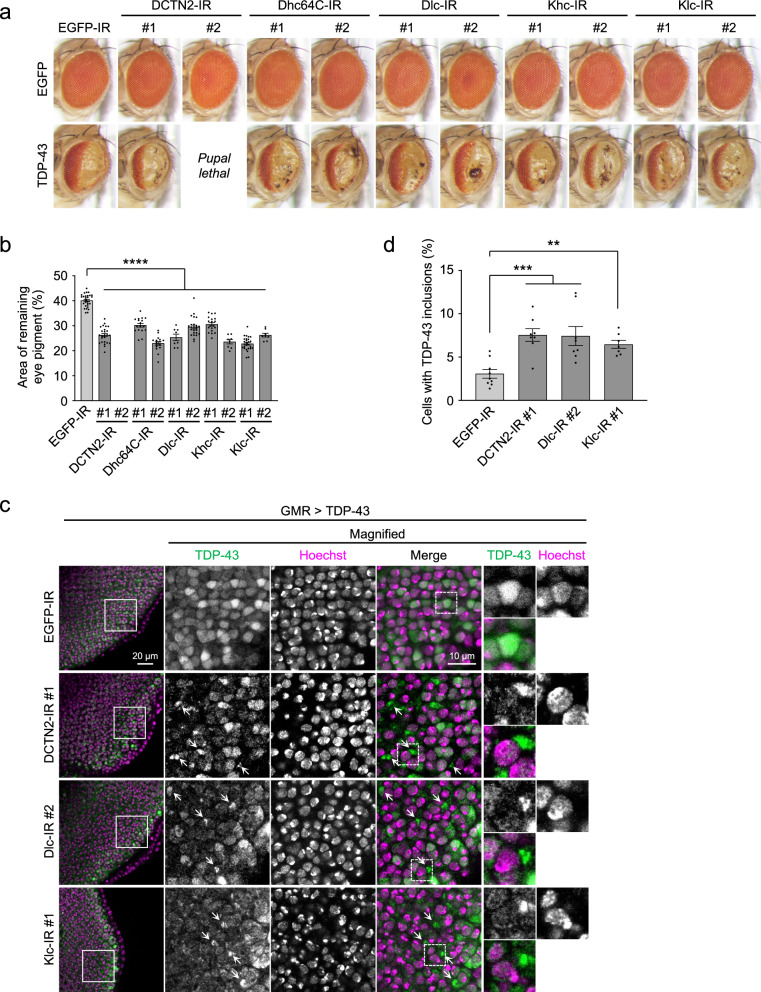
Deficiency in microtubule-associated motor proteins exacerbates TDP-43 pathology in *Drosophila.* **a** Light microscopic images of the compound eyes of EGFP flies or TDP-43 flies coexpressing EGFP-IR or inverted repeat RNAs against DCTN2 (p50, dynamitin), dynein heavy chain (Dhc64C), dynein light chain (Dlc), kinesin heavy chain (Khc), or kinesin light chain (Klc), under the control of the GMR-Gal4 driver. TDP-43 flies coexpressing DCTN2-IR#2 were not analyzed due to their lethality at the pupal stage. **b** Bar graph showing the area of remaining eye pigment of TDP-43 flies in **a**. Each dot represents data from each fly (*n* = 8–26 flies). **c** Confocal microscopic images of TDP-43 antibody staining of larval eye discs of TDP-43 flies coexpressing EGFP-IR, dDCTN2-IR#1, Dlc-IR#2, or Klc-IR#1. Arrows indicate cytoplasmic inclusions of TDP-43. Hoechst 33342 was used for nuclear staining. Scale bars, 20 µm (left) and 10 μm (right). **d** Bar graph showing the ratio of cells with cytoplasmic inclusions of TDP-43 in (**c**). Data are presented as the mean ± SEM of 7–9 fly larvae. Statistical analyses in **b** and **d** were performed to assess differences from the EGFP-IR-expressing TDP-43 flies by one-way ANOVA followed by the Dunnett multiple comparison test (**p < 0.01, ***p < 0.001, ****p < 0.0001)

Because DCTN1 is a component of dynactin, a multiprotein complex functioning in intracellular transport along microtubules together with the cytoskeletal motor protein dynein, the exacerbation of TDP-43 aggregation in TDP-43 flies coexpressing dDCTN1-IR might be attributed to the impairment of microtubule-dependent transport. To test this hypothesis, we next analyzed whether knockdown of other components of microtubule-associated motor protein complexes would affect TDP-43-mediated toxicity in the TDP-43 flies. We found that knockdown of DCTN2 (p50, dynamitin), a component of dynactin, by coexpressing dDCTN2-IR #1 in the TDP-43 flies exacerbated loss of pigmentation in the compound eyes (Fig. 5a, b), similar to the results of DCTN1 knockdown. The TDP-43-mediated eye degeneration was also exacerbated by the knockdown of dynein heavy chain Dhc64c and dynein light chain Dlc (Fig. 5a, b), suggesting that the impairment of the dynein/dynactin complex enhances TDP-43 toxicity. In addition, knockdown of kinesin heavy chain Khc and kinesin light chain Klc, components of kinesin, another microtubule-associated motor protein complex, also exacerbated the eye degeneration of TDP-43 flies (Fig. 5a, b). These results indicate that knockdown of microtubule-associated motor proteins commonly exacerbates TDP-43-mediated neurodegeneration. We next performed immunohistochemical analyses of the larval eye discs of these TDP-43 flies, and found that the knockdown of microtubule-associated motor proteins, including DCTN2, Dlc, and Klc, leads to the cytoplasmic mislocalization of TDP-43 and promotes the formation of cytoplasmic inclusions of TDP-43 in TDP-43 flies (Fig. 5c, d), similar to the results of DCTN1 knockdown (Fig. 2). These results indicate that deficiency in microtubule-associated motor proteins exacerbates TDP-43 pathology in vivo, possibly through the dysfunction of intracellular transport along microtubules.

## Discussion

Although abnormal aggregation of TDP-43 into inclusions is a common pathological hallmark of ALS/FTD, what drives the aggregation of TDP-43 remains unclear. In the present study, we addressed this issue by investigating the role of DCTN1, a causative gene of Perry syndrome in which patients develop TDP-43 pathology, in the formation of TDP-43 aggregation using a *Drosophila* model of ALS/FTD. We demonstrated in vivo that the knockdown of DCTN1, as well as other components of microtubule-associated motor protein complexes, including dynactin, dynein, and kinesin, exacerbates pathological TDP-43 aggregation in the cytoplasm. Cell culture experiments demonstrated that DCTN1 knockdown delays the disassembly of stress granules and promotes the formation of ubiquitin-positive TDP-43 inclusions in stressed cells. These results demonstrate that a deficiency in DCTN1 and other microtubule-associated motor proteins drives the aggregation of wild-type TDP-43 through the dysregulation of stress granule dynamics, indicating the crucial role of intracellular transport along microtubules in the pathological development of the TDP-43 proteinopathies, including ALS/FTD.

Our work provides important insights into the mechanisms of TDP-43 aggregation, and a potential therapeutic target for the TDP-43 proteinopathies. First, dysfunction of microtubule-dependent processes drives TDP-43 pathology in vivo. We demonstrated using ALS/FTD flies that DCTN1 knockdown not only exacerbates the neurodegeneration caused by TDP-43, but also promotes the formation of ubiquitin-positive inclusions of TDP-43 in the cytoplasm (Figs. 1, 2). DCTN1 loss and disease-linked mutations in DCTN1 alone were previously shown to cause neuronal dysfunction and eventual neurodegeneration in cultured neurons [29], *C. elegans* [16], *Drosophila* [2, 15, 23], and mice [7, 26, 45]. Our data showing the appearance of abnormal small vacuole-like structures in the retina of DCTN1-IR flies (Fig. 1d) is in line with these previous reports; the toxicity of DCTN1 loss was not detectable on light microscopy (Fig. 1b) or the climbing assay (Fig. 1e) in the flies expressing DCTN1-IR alone, possibly owing to the sensitivity of the experimental methods and the low knockdown efficiency in our flies. Although several reports have suggested that DCTN1 deficiency itself potentially causes detrimental effects on cell survival, it was unknown whether DCTN1 genetically interacts with TDP-43 pathology; therefore, our study is the first report to our knowledge demonstrating that DCTN1 is a genetic modifier that promotes TDP-43 aggregation in vivo. DCTN1 is a subunit of dynactin, a motor protein complex functioning in microtubules. Intriguingly, knockdown of other components of microtubule-associated motor protein complexes, such as dynein and kinesin, also exacerbated the cytoplasmic aggregation of TDP-43 in ALS/FTD flies (Fig. 5). These data indicate that intracellular transport along microtubules plays a crucial role in the development of TDP-43 pathology. In support of this, mutations in *TUBA4A* were recently identified as the genetic cause of familial FTD with TDP-43 pathology [28]. *TUBA4A* encodes α-tubulin, a major component of the microtubule network, and FTD patients with *TUBA4A* mutations demonstrated a decreased trend of α-tubulin levels and impairment of microtubule network reformation, implying that dysfunction of microtubule-dependent cellular activities triggers TDP-43 pathology in patients. Considering that microtubule-dependent transport is likely impaired by various stresses and aging [11, 38], our results highlight the importance of abnormalities in microtubule functions in the TDP-43 proteinopathies, including ALS/FTD.

Second, stress granule dynamics is regulated via microtubule-dependent processes in heat-stressed cells. We showed that DCTN1 deficiency does not affect the formation of but delays the disassembly of stress granules during recovery after stress (Fig. 3). Nocodazole treatment also delayed stress granule disassembly (Additional file 1: Fig. SI–2). These results suggest that intracellular transport along microtubules is necessary for the disassembly of stress granules. Importantly, DCTN1 knockdown facilitated the formation of ubiquitin-positive cytoplasmic TDP-43 aggregates in the heat-stressed cells (Fig. 4). Taken together, we propose the following model for TDP-43 aggregation: upon stress, TDP-43, together with other RNA-binding proteins and RNAs, transiently forms stress granules in the cytoplasm, which are readily disassembled in a manner that depends on intracellular transport along microtubules. Mutations in DCTN1, or dysfunction in intracellular transport along microtubules, cause the dysregulation of stress granule dynamics, which leads to the aberrant aggregation of stress granule components, including TDP-43 (Additional file 1: Fig. SI–3). Previous studies reported that degradation machineries, such as autophagy and the proteasome, the molecular chaperone Hsp70, and ubiquitination are required for the disassembly of stress granules [3, 12, 25, 40]. Therefore, functional decline not only in microtubule-dependent transport, but also in multiple cellular processes that regulate the formation and disassembly of stress granules, may lead to the formation of TDP-43 aggregates through the impairment of stress granule dynamics. Although it remains to be clarified as to how stress granule components are sorted and cleared during recovery after stress, and how DCTN1 deficiency delays the disassembly of stress granules and promotes pathological TDP-43 aggregation, abnormalities in microtubule functions may cause impaired transport of stress granule components, leading to aberrant aggregation of TDP-43 and other components.

Finally, intracellular transport along microtubules might be a therapeutic target for the TDP-43 proteinopathies. Although it remains unclear as to how TDP-43 aggregation causes neurodegeneration in patients with ALS/FTD, suppressing TDP-43 aggregation will potentially rescue neurons from TDP-43 toxicity. Indeed, we recently showed that a transient reduction in TDP-43 levels by antisense oligonucleotides leads to the suppression of cytoplasmic TDP-43 aggregation, and long-lasting improvement in behavioral abnormalities in ALS/FTD mice [36]. In the present study, we showed that knockdown of DCTN1 and other microtubule-dependent motor proteins facilitates TDP-43 aggregation and exacerbates neurodegeneration in vivo (Figs. 1, 2, 5). Our data indicate that the impairment of intracellular transport along microtubules is a key modifier that drives TDP-43 aggregation in the TDP-43 proteinopathies, indicating the possibility that the restoration of microtubule functions may prevent aberrant TDP-43 aggregation and neurodegeneration. Microtubule stability and functions are regulated by the post-translational modifications of tubulins, including acetylation. Promoting the acetylation of α-tubulin by the inhibition of histone deacetylase 6 (HDAC6), a major deacetylating enzyme of α-tubulin, was shown to normalize microtubule-dependent axonal transport in mouse models of Charcot-Marie-Tooth disease with HSPB1 mutations [6], and in motor neurons derived from ALS patients with familial mutations in FUS and TDP-43 [10, 14]. Thus, the reactivation of microtubule functions through HDAC6 inhibition might reverse not only defects in microtubule-dependent transport, but also the accumulation of TDP-43 aggregates, providing a potential therapeutic approach for the TDP-43 proteinopathies, including ALS/FTD.

## Materials and methods

### Fly stocks

Flies were cultured and crossed under standard conditions at 25 °C. Transgenic flies bearing the UAS-TDP-43 transgene have been previously described [17]. Transgenic flies bearing the UAS-dDCTN1-IR (#1, 24760; #2, 24761) and UAS-grim (9922) transgenes were obtained from the Bloomington *Drosophila* Stock Center, and transgenic flies bearing the UAS-DCTN2-IR (#1, 23726; #2, 110741), UAS-Dhc64C-IR (#1, 28053; #2, 28054), UAS-Dlc-IR (#1, 22760; #2, 105760), UAS-Khc-IR (#1, 44337; #2, 44338), and UAS-Klc-IR (#1, 22125; #2, 39583) transgenes were obtained from the Vienna *Drosophila* Resource Center. Female flies were used in all experiments.

### Fly eye imaging

The eyes of one-day-old flies were analyzed and imaged using the stereomicroscope SZX10 equipped with the digital camera DP22 (Olympus). Areas of eye pigmentation and necrotic patches were analyzed using ImageJ software (NIH). For the histological analysis of retinal structures, fly heads (1-day-old) were fixed in Carnoy’s solution, and embedded in paraffin. Eye sections of 3-µm thickness were stained with hematoxylin and eosin, and analyzed using the microscope BX51 with a CCD camera DP71 (Olympus).

### Quantitative RT-PCR

The expression of DCTN1 in neurons and other tissues in *Drosophila* has been reported previously [8]. Total RNA was isolated from adult flies (five flies per sample) using the RNeasy Lipid Tissue Mini Kit (Qiagen). cDNA was synthesized from total RNA using the PrimeScript RT Master Mix (Qiagen), and real-time PCR was performed using the Mx3000P qPCR System (Agilent) and Premix Ex Taq (Takara Bio). The primer sequences were as follows:

*dDCTN1* forward: 5′-AGCCGTGCCAGGTTTG-3′

*dDCTN1* reverse: 5′-CGTTCGCCCTCATACA-3′

*rp49* forward: 5′-AGCGCACCAAGCACTTCATCCGCCA-3′

*rp49* reverse: 5′-GCGCACGTTGTGCACCAGGAACTTC-3′

### Climbing assay

The climbing assay was performed as previously described [35]. Briefly, flies at 7 days of age in a glass vial were gently tapped to the bottom of the vial, and the height of each fly after 10 s of climbing was scored as follows: 0 (less than 2 cm), 1 (between 2 and 3.9 cm), 2 (between 4 and 5.9 cm), 3 (between 6 and 7.9 cm), 4 (between 8 and 9.9 cm), and 5 (more than 10 cm). In each experiment, the trial was repeated five times at 20 s intervals, and the scores were averaged (> 30 flies).

### Immunohistochemistry

Immunohistochemistry was performed as previously described [17]. Briefly, eye imaginal discs of third instar larvae were fixed with 4% paraformaldehyde (PFA) and then incubated overnight at 4 °C with the following antibodies: anti-TDP-43 (12892–1-AP rabbit polyclonal, Proteintech), and anti-ubiquitin (FK2 mouse monoclonal, MBL). As secondary antibodies, Alexa Fluor-labeled IgGs (Thermo Fisher Scientific) were used. Hoechst 33342 was used for nuclear staining. Images were taken with the confocal laser-scanning microscope FV3000 (Olympus). Cytoplasmic inclusions of TDP-43 were analyzed using ImageJ software (NIH). Cytoplasmic inclusions were defined as inclusions with strong fluorescence signals that are localized around Hoechst 33342 nuclear staining, but do not overlap with the Hoechst staining.

### Detergent solubility assay

Five adult fly heads were lysed with a lysis buffer containing 1% Triton X-100 supplemented with protease inhibitors (Nacalai Tesque), and centrifuged at 20,000 × g for 10 min at 4 °C to separate the supernatant (S1) and pellet fractions. The pellets (insoluble in Triton X-100) were suspended in a buffer containing 1% sarkosyl, sonicated, and centrifuged at 20,000 × *g* for 10 min at 4 °C. The supernatants were removed as the sarkosyl-soluble fraction S2, and the pellets were solubilized in 9 M urea (the sarkosyl-insoluble fraction P2).

### FCS measurements

Ten adult fly heads were lysed in a lysis buffer containing 0.5% Triton X-100 supplemented with protease inhibitors (Nacalai Tesque), and were centrifuged at 10,000 × *g* for 30 min at 4 °C. The supernatant fractions were subjected to FCS measurements. FCS measurements were performed at room temperature using the FlucDEUX system with a × 40 objective lens (NA1.15, water immersion) (MBL). The autocorrelation function G(τ) was calculated and fitted by FCCS Editor software (MBL) using Eq. 1,

1 $$\text{G(}\tau \text{) = }\frac{\langle I\left(t\right)I(t+\tau )\rangle }{{\langle I(t)\rangle }^{2}}=1+\frac{1}{N}{(1+\frac{\tau }{{\tau }_{i}})}^{-1}{(1+\frac{\tau }{{s}^{2}{\tau }_{i}})}^{-\frac{1}{2}}$$

where < *I(t)* > is the average fluorescence intensity (photons per second), *N* is the number of particles in the detection area, and *s* is the structural parameter based on the dimensions of the confocal volume. The correlation time (τ_*i*_), which is the average time for diffusion of fluorescent particles across the detection area, correlates with the size of the fluorescent particles.

### Cell culture, transfection, and immunocytochemistry

Human embryonic kidney 293 (HEK293) cells were cultured in DMEM supplemented with 10% fetal bovine serum at 37 °C under 5% CO_2_. For heat shock, cells were incubated at 42 °C for 60 min. For RNAi-mediated knockdown, cells were transfected with siRNA against the human DCTN1 gene or nontargeting control siRNA (Dharmacon) using Lipofectamine RNAiMAX (Invitrogen), and incubated for 48 h. For immunocytochemistry, cells were fixed with 4% PFA, permeabilized with 0.3% Triton X-100, and incubated with the following antibodies: anti-TDP-43 (12892–1-AP rabbit polyclonal, Proteintech), anti-G3BP1 (611127 mouse monoclonal, BD), and anti-ubiquitin (FK2 mouse monoclonal, MBL). As secondary antibodies, Alexa Fluor-labeled IgGs were used. Hoechst 33342 was used for nuclear staining. Images were taken with the confocal laser-scanning microscope FV3000. Cells with TDP-43- and G3BP1-positive dots (stress granules), and TDP-43 inclusions with ubiquitin staining (TDP-43 aggregates) were analyzed using ImageJ software.

### Western blot analysis

Proteins were separated using 5–20% gradient sodium dodecyl sulfate–polyacrylamide gel electrophoresis gels (Atto), and transferred onto polyvinylidene fluoride membranes (Bio-Rad). Membranes were incubated overnight at 4 °C with the following antibodies: anti-TDP-43 (10782–2-AP rabbit polyclonal, Proteintech), anti-DCTN1 (sc-365274 mouse monoclonal, Santa Cruz), and anti-actin (AC-40 mouse monoclonal, Sigma). As secondary antibodies, horseradish peroxidase (HRP)-conjugated IgGs (Jackson Immuno Research Laboratory) were used. The HRP signal was visualized using ImmunoStar Zeta (Wako), and captured using Amersham Imager 600 (Cytiva). Images were analyzed using ImageJ software.

### Statistical analyses

Data in the fly experiments, including data from quantitative RT-PCR, eye pigmentation and necrotic patch analyses, the climbing assay, and quantification of TDP-43 inclusions by immunohistochemistry, were analyzed by one-way analysis of variance (ANOVA) followed by the Dunnett multiple comparison test (Figs. 1a and c, 2b, 5b and d) or by the Tukey multiple comparison test (Fig. 1f). The data in the cell culture experiments, including densitometric analysis of the immunoblot images (Fig. 3c), quantification of stress granules (Fig. 3f and i), and TDP-43 aggregation (Fig. 4d), were analyzed by the Student *t*-test. For all analyses, GraphPad Prism software was used. A *p*-value of less than 0.05 was considered to indicate a statistically significant difference between groups.

### Supplementary Information

**Additional file 1: Figure SI-1. **Heterozygous deletion of the dDCTN1 gene also exacerbates eye degeneration in TDP-43 flies. **a** Relative levels of dDCTN1 mRNA in adult fly brains analyzed by quantitative RT-PCR. Data were normalized using *rp49* mRNA levels, and are presented as relative values to dDCTN1 mRNA levels of wild-type flies. Data are presented as the mean ± standard error of the mean (SEM) of three independent experiments. Each dot represents data from a single experiment. Fly genotypes: +/+, *Elav-Gal4*/+; Df(3L)fz-GF3b/+, *Elav-Gal4*/+;;*Df(3L)fz-GF3b*/+. **b** Light microscopic images of the compound eyes of wild-type flies and TDP-43 flies heterozygous for the dDCTN1 deletion. **c** Bar graph showing the area of remaining eye pigment of the TDP-43 flies in **b**. Each dot represents data from a single fly (n = 8–12 flies). Fly genotypes: +/+, *GMR-Gal4*/+; Df(3L)fz-GF3b/+, *GMR-Gal4*/+;;*Df(3L)fz-GF3b*/+; TDP-43/+, *GMR-Gal4*/+;;*TDP-43*/+; TDP-43/Df(3L)fz-GF3b, *GMR-Gal4*/+;;*TDP-43*/*Df(3L)fz-GF3b*. Statistical analyses in **a** and **c** were performed to assess differences between groups by the Student *t*-test (*p < 0.05, ****p < 0.0001). **Figure SI-2.** Disruption of the microtubule network by nocodazole treatment delays the disassembly of stress granules in heat-stressed cells. **a** Schematic representation of recovery experiments in the presence of nocodazole. **b**, **c** Microscopic images (**b**) and a bar graph (**c**) of stress granules in cells that were treated with nocodazole for 30 min after HS. Statistical analysis in** c** was performed to assess differences between groups by the Student *t*-test (** *p* < 0.01). Scale bars, 50 µm (top) and 10 µm (bottom) (**b**). **Figure SI-3. **Proposed mechanism of TDP-43 aggregation through the dysregulation of stress granule dynamics by impairment of DCTN1/microtubule functions. Upon stress, TDP-43, together with other RNA-binding proteins and RNAs, transiently forms stress granules in the cytoplasm (**a**), which are readily disassembled during recovery after the removal of stress (**b**). The formation and disassembly of stress granules are tightly regulated to protect cellular homeostasis against stresses, and intracellular transport along microtubules is likely required at least for the proper disassembly of stress granules. Mutations in DCTN1, or abnormalities in microtubule-dependent transport, causes the delayed disassembly of stress granules (**c**), which potentially leads to the formation of aberrant aggregates of TDP-43 in the cytoplasm (**d**).

## Data Availability

All data generated or analyzed during this study are included in this article.
