# Supplementary material for: Dysregulation of stress granule dynamics by DCTN1 deficiency exacerbates TDP-43 pathology in Drosophila models of ALS/FTD
Source: Acta Neuropathol Commun. 2024 Feb 4;12:20. doi: 10.1186/s40478-024-01729-8 (PMC10840176; doi:10.1186/s40478-024-01729-8)
Supplement: Supplementary file 1 — Additional file 1: Figure SI-1. Heterozygous deletion of the dDCTN1 gene also exacerbates eye degeneration in TDP-43 flies. a Relative levels of dDCTN1 mRNA in adult fly brains analyzed by quantitative RT-PCR. Data were normalized using rp49 mRNA levels, and are presented as relative values to dDCTN1 mRNA levels of wild-type flies. Data are presented as the mean ± standard error of the mean (SEM) of three independent experiments. Each dot represents data from a single experiment. Fly genotypes: +/+, Elav-Gal4/+; Df(3L)fz-GF3b/+, Elav-Gal4/+;;Df(3L)fz-GF3b/+. b Light microscopic images of the compound eyes of wild-type flies and TDP-43 flies heterozygous for the dDCTN1 deletion. c Bar graph showing the area of remaining eye pigment of the TDP-43 flies in b. Each dot represents data from a single fly (n = 8–12 flies). Fly genotypes: +/+, GMR-Gal4/+; Df(3L)fz-GF3b/+, GMR-Gal4/+;;Df(3L)fz-GF3b/+; TDP-43/+, GMR-Gal4/+;;TDP-43/+; TDP-43/Df(3L)fz-GF3b, GMR-Gal4/+;;TDP-43/Df(3L)fz-GF3b. Statistical analyses in a and c were performed to assess differences between groups by the Student t-test (*p < 0.05, ****p < 0.0001). Figure SI-2. Disruption of the microtubule network by nocodazole treatment delays the disassembly of stress granules in heat-stressed cells. a Schematic representation of recovery experiments in the presence of nocodazole. b, c Microscopic images (b) and a bar graph (c) of stress granules in cells that were treated with nocodazole for 30 min after HS. Statistical analysis in c was performed to assess differences between groups by the Student t-test (** p < 0.01). Scale bars, 50 µm (top) and 10 µm (bottom) (b). Figure SI-3. Proposed mechanism of TDP-43 aggregation through the dysregulation of stress granule dynamics by impairment of DCTN1/microtubule functions. Upon stress, TDP-43, together with other RNA-binding proteins and RNAs, transiently forms stress granules in the cytoplasm (a), which are readily disassembled during recovery after the removal of stress [file 40478_2024_1729_MOESM1_ESM.pdf]

Supplementary Information Figure SI-1

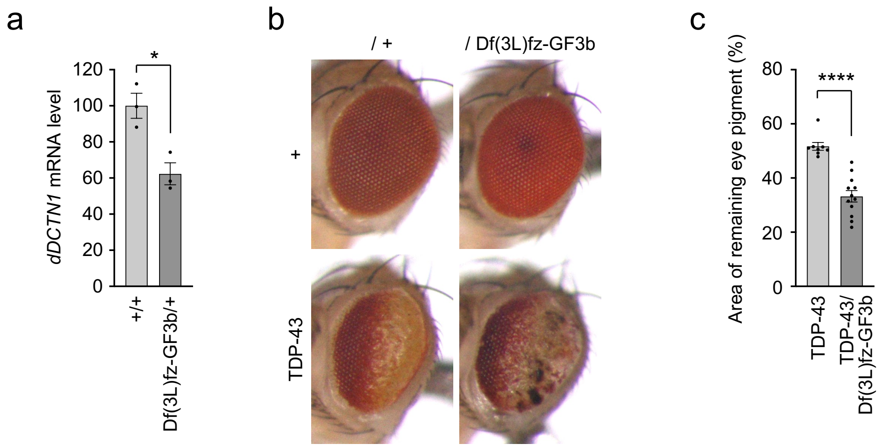

Fig. SI-1 Heterozygous deletion of dDCTN1 gene also exacerbates eye degeneration in TDP-43 flies. (**a**) Relative levels of dDCTN1 mRNA in adult fly brains analyzed by quantitative RT-PCR. Data were normalized using *rp49* mRNA levels, and are presented as relative values to dDCTN1 mRNA levels of wild-type flies. Data are presented as the mean ± standard error of the mean (SEM) of three independent experiments. Each dot represents data from a single experiment. Fly genotypes: +/+, *Elav-Gal4*/+; Df(3L)fz-GF3b/+, *Elav-Gal4*/+;;*Df(3L)fz-GF3b*/+. (**b**) Light microscopic images of the compound eyes of wild-type flies and TDP-43 flies heterozygous for the dDCTN1 deletion. (**c**) Bar graph showing the area of remaining eye pigment of the TDP-43 flies in (**b**). Each dot represents data from a single fly (n = 8–12 flies). Fly genotypes: +/+, *GMR-Gal4*/+; Df(3L)fz-GF3b/+, *GMR-Gal4*/+;;*Df(3L)fz-GF3b*/+; TDP-43/+, *GMR-Gal4*/+;;*TDP-43*/+; TDP-43/Df(3L)fz-GF3b, *GMR-Gal4*/+;;*TDP-43*/*Df(3L)fz-GF3b*. Statistical analyses in (**a**) and (**c**) were performed to assess differences between groups by the Student *t*-test (*$p < 0.05$, ****$p < 0.0001$).

Supplementary Information Figure SI-2

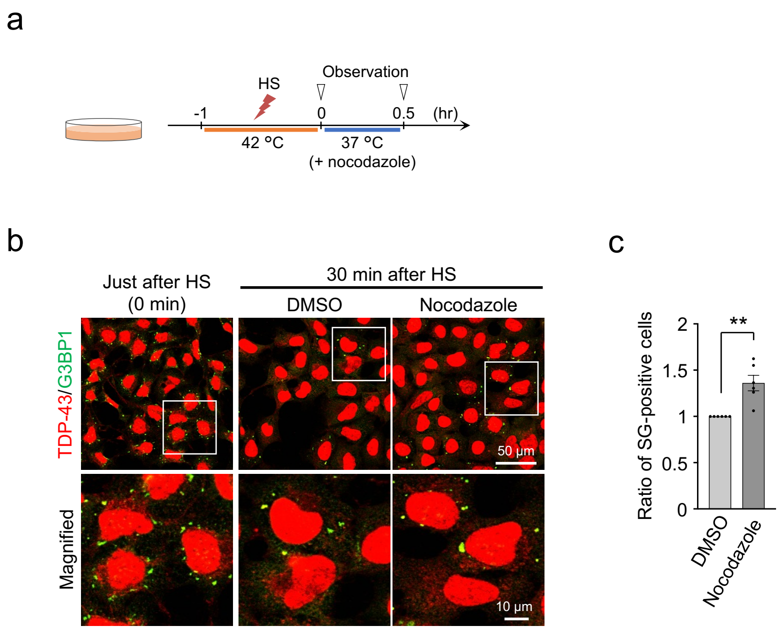

Fig. SI-2 Disruption of the microtubule network by nocodazole treatment delays the disassembly of stress granules in heat-stressed cells. (**a**) Schematic representation of recovery experiments in the presence of nocodazole. (**b, c**) Microscopic images (**b**) and a bar graph (**c**) of stress granules in cells that were treated with nocodazole for 30 min after HS. Statistical analysis in (**c**) was performed to assess differences between groups by the Student *t*-test (**$p < 0.01$). Scale bars, 50 µm (top) and 10 µm (bottom) (**b**)

Supplementary Information Figure SI-3

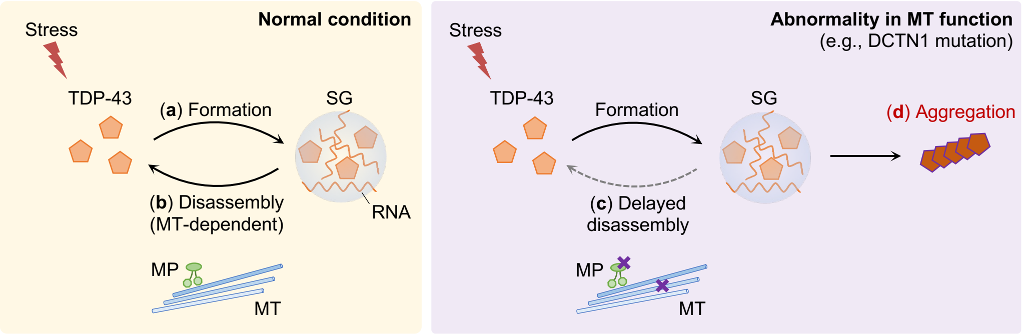

SG: stress granule; MT: microtubule; MP: motor protein

Fig. SI-3 Proposed mechanism of TDP-43 aggregation through the dysregulation of stress granule dynamics by impairment of DCTN1/microtubule functions. Upon stress, TDP-43, together with other RNA-binding proteins and RNAs, transiently forms stress granules in the cytoplasm (**a**), which are readily disassembled during recovery after the removal of stress (**b**). The formation and disassembly of stress granules are tightly regulated to protect cellular homeostasis against stresses, and intracellular transport along microtubules is likely required at least for the proper disassembly of stress granules. Mutations in DCTN1, or abnormalities in microtubule-dependent transport, causes the delayed disassembly of stress granules (**c**), which potentially leads to the formation of aberrant aggregates of TDP-43 in the cytoplasm (**d**).
